# Influence of atherosclerosis risk factors on the anatomical distribution of peripheral arterial disease in patients with chronic limb-threatening ischemia: a cross-sectional study

**DOI:** 10.1590/1677-5449.202300141

**Published:** 2023-07-17

**Authors:** Vanessa Prado dos Santos, Camila Izabel Cerutti, Marcelo José Carlos Alencar, André Brito Queiroz, Lucas de Mello Ferreira, Cícero Fidelis, José Siqueira de Araújo, Carlos Alberto Silveira Alves

**Affiliations:** 1 Universidade Federal da Bahia - UFBA, Hospital Universitário Professor Edgard Santos - HUPES, Salvador, BA, Brasil.

**Keywords:** atherosclerosis, chronic limb-threatening ischemia, peripheral arterial disease, diabetes mellitus, risk factors

## Abstract

**Background:**

Atherosclerosis risk factors can have different impacts on cardiovascular diseases and on the anatomical distribution of Peripheral Arterial Disease (PAD).

**Objectives:**

To study the influence of atherosclerosis risk factors on the anatomical distribution of PAD in patients with chronic limb-threatening ischemia (CLTI).

**Methods:**

We performed an observational, cross-sectional, and analytical study that included 476 hospitalized patients with CLTI due to PAD. We compared the presence of atherosclerosis risk factors (age, gender, diabetes mellitus, smoking, and hypertension) in patients with PAD involving three different anatomic areas (aortoiliac, femoropopliteal, and infrapopliteal). Multivariate analysis was performed to identify associations between atherosclerosis risk factors and PAD distribution.

**Results:**

The mean age of the 476 patients was 69 years, 249 (52%) were men, and 273 (57%) had diabetes. Seventy-four percent (353) had minor tissue loss. Multivariate analysis identified three risk factors associated with PAD anatomical distribution (gender, smoking, and DM). Women had a 2.7 (CI: 1.75-4.26) times greater chance of having femoropopliteal disease. Smokers had a 3.6-fold (CI: 1.54-8.30) greater risk of aortoiliac disease. Diabetic patients were 1.8 (CI: 1.04-3.19) times more likely to have isolated infrapopliteal occlusive disease.

**Conclusions:**

The study showed that gender, DM, and smoking impact on the anatomical distribution of PAD in patients with CLTI. Diabetic patients were more likely to have only infrapopliteal disease, women had a greater risk of femoropopliteal PAD, and smokers had a greater risk of aortoiliac occlusive disease.

## INTRODUCTION

Cardiovascular diseases (CVD) are the principal cause of mortality in Brazil and worldwide.^1^ Atherosclerosis is the major cause of CVD and the three most important diagnoses related to the disease are ischemic cardiac disease, cerebral vascular accident (stroke), and peripheral arterial disease (PAD).^2^ Globally, there was a 71.5% increase in absolute numbers of new cases of PAD from 1990 to 2019, reaching 10,504,092 cases in 2019.^3^ However, the age-adjusted prevalence of PAD revealed a 21.7% decline from 1990 to 2019, primarily in countries with high income and high sociodemographic indices.^3^ In the United Kingdom, the incidence and prevalence of symptomatic PAD fell from 2000 to 2014, with a drop in prevalence from 3.4 to 2.4%.^4^ Brazil is one of a group of countries with estimated age-adjusted PAD incidence of less than 100 people per 100 thousand inhabitants.^3^ The prevalence of PAD increases with age in both sexes, with highest incidence in the age range of 90-94 years for men, and 75-79 years for women.^3^ In general, above 40 years of age, PAD prevalence and incidence rates tend to be higher among women than men.^3^

Advanced age, diabetes mellitus (DM), systemic arterial hypertension (SAH), and smoking are factors that are commonly associated with risk of CVD and PAD, for which atherosclerosis is the principal etiology.^5^ Considering the behavior of PAD risk factors, from 2010 to 2019, there was a reduction in exposure to smoking and a greater than 1% per year increase in exposure to hyperglycemia globally.^6^ Globally, the risk of exposure to hyperglycemia increase significantly, by 1.32% (1.01 to 1.64) a year, while smoking reduced by 1.20% (-1.29 to -1.11).^6^ When compared to hyperglycemia, the increase in exposure to arterial hypertension was smaller, at 0.51% (0.04 to 1.00) per year, from 2010 to 2019.^6^ The prevalence of SAH is 32.3% in the adult Brazilian population, considering blood pressure measured by instruments and/or patients taking antihypertensive medication, and prevalence increases with age, reaching 71.7% in the over 70s.^7^

Diabetes mellitus constitutes a severe public health problem all over the world.^8^ Over the years, the prevalence of DM has been increasing in many different countries.^9,10^ In the United States, estimated prevalence was 9.5% in the period from 1999 to 2002, reaching 12% between 2013 and 2016.^9^ In 2018, it was estimated that there were more than 34 million people with DM, at an estimated prevalence of 13% of the adult population, rising to 26.6% among people 65 or older.^9^ In Brazil, the estimated prevalence of DM is 9.2%, with a marked difference between the country’s different regions, with rates of 6.3% in the North, 7.2% in the South, 7.6% in the Mid-West, 12.2% in the Northeast, and 12.8% in the Southeast.^10^

Diabetes and PAD contribute to higher risk of lower limb amputations in patients with ulcers and necrotic lesions.^11,12^ Patients with PAD who have Critical Limb-threatening Ischemia (CLTI) have pain at rest and gangrene or ulcers of the lower limb with duration exceeding 2 weeks, characterizing advanced disease and increased risk of limb loss.^12,13^ It is estimated that symptoms of critical limb ischemia are the initial clinical presentation in 1 to 3% of PAD patients, but the epidemiology of CLTI has not been clearly established.^12,13^ In the United States, when the estimated prevalence of PAD was 10.69%, the prevalence of critical limb ischemia was 1.33%, but was higher among diabetics and patients who had had strokes or heart failure.^14^ Also in the United States, more than 130,000 hospital admissions of diabetic patients during 2016 were related to amputations.^9^ Worldwide, around 6.8 million people underwent amputations related to complications of DM in 2016.^11^

Considering the prevalence and morbidity of PAD, of CLTI, and of their risk factors, the objective of this study was to identify the influence of different risk factors for atherosclerosis on the anatomic distribution of PAD among patients with chronic limb-threatening ischemia. The study hypothesis is that the different risk factors influence on the anatomic distribution of the proximal obstruction level in PAD.

## METHODS

**Figure 1:**
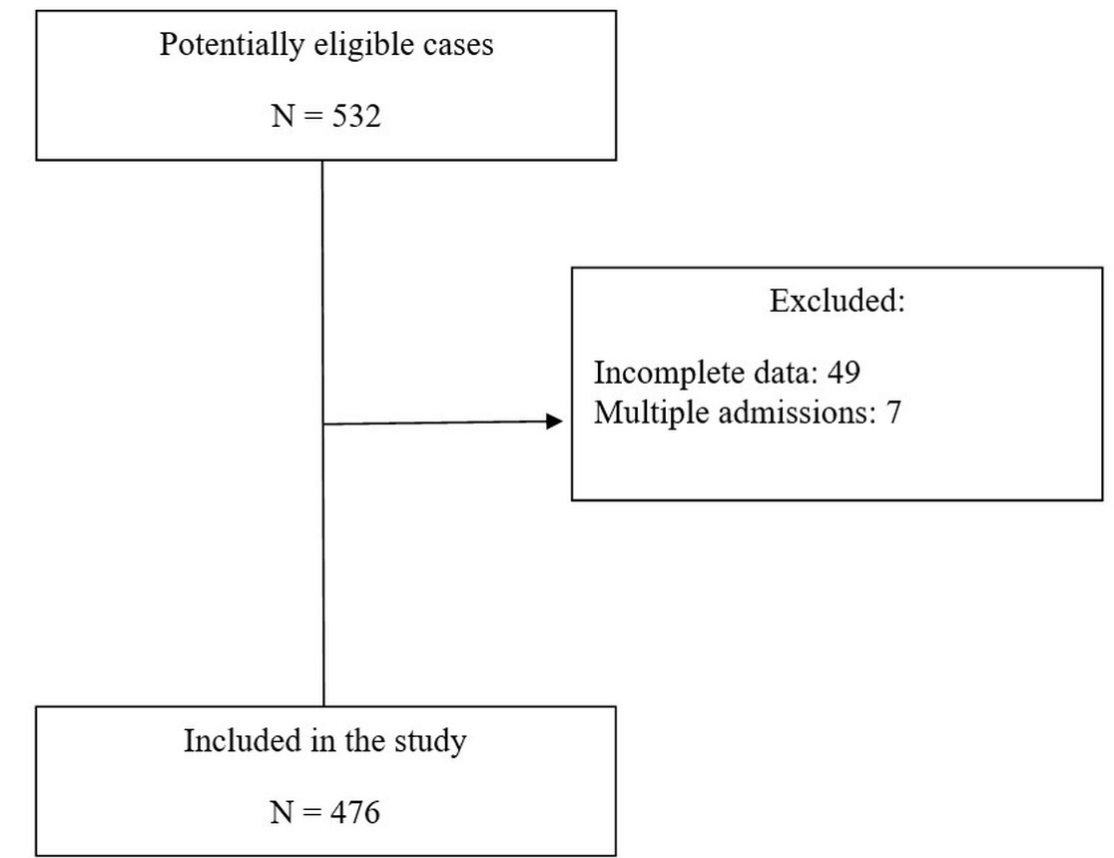
Flow diagram of selection, exclusion, and inclusion of the cases analyzed.

A cross-sectional, retrospective, observational study was conducted, consecutively enrolling patients with a diagnosis of chronic critical lower limb ischemia secondary to PAD admitted from 2014 to 2017 to the Professor Edgard Santos University Hospital (HUPES), run by the Universidade Federal da Bahia (UFBA). Since this was an observational study, the Strengthening the Reporting of Observational Studies in Epidemiology^15^ recommendations were used to describe it. The study recruited a total of 476 patients consecutively admitted for treatment of CLTI secondary to PAD of atherosclerotic etiology. The sample size calculation was based on an alpha error of 0.05, study power of 80%, and a systematic review of the literature^16^ on the proportion of PAD in three different anatomic areas (aortoiliac, femoropopliteal, and tibial/infrapopliteal) in people with and without DM. The calculation estimated a minimum sample size of 114 patients for analysis of the difference between people with and without DM and aortoiliac disease, 408 cases for femoropopliteal involvement, and 242 patients for infra-popliteal disease. Figure 1 shows a flow chart illustrating inclusion of cases. The research protocol was approved by the Research Ethics Committee at the same institution, under decision number 2,441,556.

Data were collected by review of archived medical records, clinical charts, and imaging exams from patients admitted with diagnoses of CLTI by the vascular surgery service at the Professor Edgard Santos University Hospital (HUPES). All of the patients enrolled had pain at rest and gangrene (with major or minor tissue loss) or ulcers with duration exceeding 2 months. Risk factors for atherosclerosis (sex, age, SAH, DM, and smoking) were studied together with their possible associations with the anatomic location of PAD (aortoiliac, femoropopliteal, or infrapopliteal) identified during physical examination and confirmed with imaging methods. Patients were divided into 3 PAD anatomic distribution groups on the basis of physical examination of pulses and imaging exams (aortoiliac, femoropopliteal, or infrapopliteal), indicating the site of proximal obstruction of the arterial tree. Patients with no femoral pulse in the limb were considered as having aortoiliac PAD. Patients with normal femoral pulses but without popliteal pulses in involved limb were considered to have femoropopliteal disease. Cases with normal femoral and popliteal pulses, but absent pedal and posterior tibial pulses were considered to have infrapopliteal disease. Absence of pulses in the cases in the sample demonstrated that there was chronic arterial occlusion or critical stenosis of the proximal arterial segment, which was then confirmed with imaging exams. All of the patients enrolled on the study had absence of both pulses at the level of the foot (pedal and posterior tibial pulses) on physical examination. All of the patients underwent imaging exams that confirmed peripheral arterial occlusive disease and its location (duplex scan, angiotomography, and/or digital subtraction angiography). When reviewing the medical records and imaging reports from the cases enrolled, descriptions of the presence of flow or opacification in all three arteries of the leg (anterior tibial, posterior tibial, and fibular) could only be found for 30 patients in the sample (6.3%), showing that the majority had multisegmental disease, with involvement of infrapopliteal arteries. Although the ankle-brachial index is routinely calculated at this service, it was not analyzed in this study.

The 3 groups of patients, distributed according to the level of proximal PAD obstruction, were compared for the presence of risk factors for atherosclerosis (age, sex, DM, SAH, and smoking). Patients were considered diabetic if they had a prior diagnosis of the disease and were being treated for it. The same criterion was used for SAH. History of heart disease and chronic kidney disease were recorded according to notes on the medical record mentioning a diagnosis, in the patient’s clinical history, or in the records of the treating professionals. Considering the retrospective nature of the study, based on review of medical records, smoking was registered if the individual had been identified as a smoker at hospital admission.

The research data were verified using Epi-Info^TM^, version 7.2.2.6. Descriptive analysis was performed for categorical and continuous variables. Associations between categorical variables (the risk factors sex, DM, SAH, and smoking) and anatomic distribution of PAD (aortoiliac, femoropopliteal, or infrapopliteal) were analyzed with chi-square test univariate analysis. Means for continuous variables, such as age, were compared with analysis of variance (ANOVA). Multivariate analysis with logistic regression was performed to test for possible associations between the 5 risk factors for atherosclerosis and the pattern of anatomic distribution of PAD. In the multivariate analysis, the anatomic distribution of PAD (the level of proximal obstruction) was analyzed as a binomial variable (aortoiliac vs. not aortoiliac; femoropopliteal vs. not femoropopliteal; and infrapopliteal vs. not infrapopliteal). A 5% significance level (p < 0.05) was adopted for rejection of the null hypothesis.

## RESULTS

Four hundred and seventy-six (476) patients were enrolled on the study. The mean age of the patients was 69 (±10 years); 249 (52%) were men, 273 (57%) were diabetic, 367 (77%) were hypertensive, and 310 (65%) were smokers. The anatomic distribution of proximal PAD obstruction, defined according to lower limb pulses and confirmed with imaging methods, was as follows: 63 (13%) patients had proximal obstruction in the aortoiliac area, 326 (69%) had femoropopliteal occlusion, with absence of popliteal and distal pulses on physical examination, and the remaining 87 (18%) only had infrapopliteal involvement, with normal popliteal pulses and absent distal pulses. The level of proximal obstruction was confirmed with imaging methods and none of the patients had pulses at the level of the ankle. The Rutherford classification^17^ for chronic ischemia was category 5 in 353 (74%) limbs and category 6 in 81 (17%), confirming tissue loss in 434 (91%) of the sample. The descriptive analysis of the sample characteristics is shown in Table 1.

**Table 1:** Characteristics of the sample (476 patients) enrolled with peripheral arterial disease and critical lower limb ischemia.

Sample characteristics (N = 476)		N (%)
Men		249 (52%)
Age (mean/years)		69 (±10) years
Smoking		310 (65%)
Diabetes mellitus		273 (57%)
Arterial hypertension		367 (77%)
History of heart disease		103 (22%)
Chronic kidney disease		34 (7%)
Time since onset of trophic lesion (mean/days)		98 (±76) days
Rutherford classification	Category 4	42 (9%)
	Category 5	353 (74%)
	Category 6	81 (17%)
Anatomic distribution of PAD^a^	Aortoiliac	63 (13%)
	Femoropopliteal	326 (69%)
	Infrapopliteal	87 (18%)

a Peripheral arterial disease.

**Figure 2:**
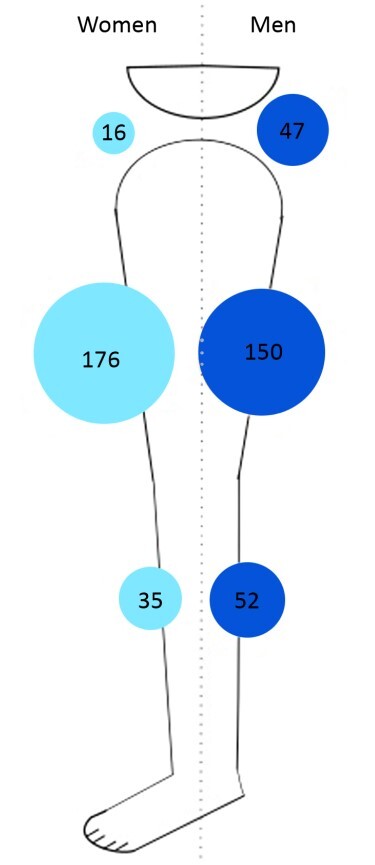
illustration of the anatomic distribution of peripheral arterial disease (aortoiliac, femoropopliteal, or infrapopliteal) by gender (men vs. women).

**Figure 3:**
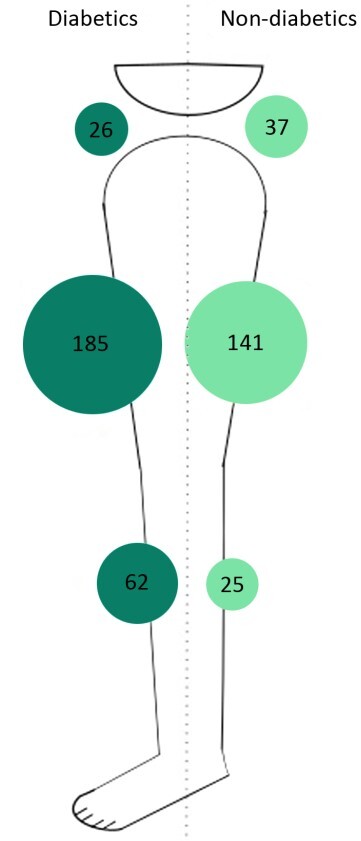
Ilustration of the anatomic distribution of peripheral arterial disease (aortoiliac, femoropopliteal, or infrapopliteal) in patients with and without diabetes mellitus (DM).

**Figure 4:**
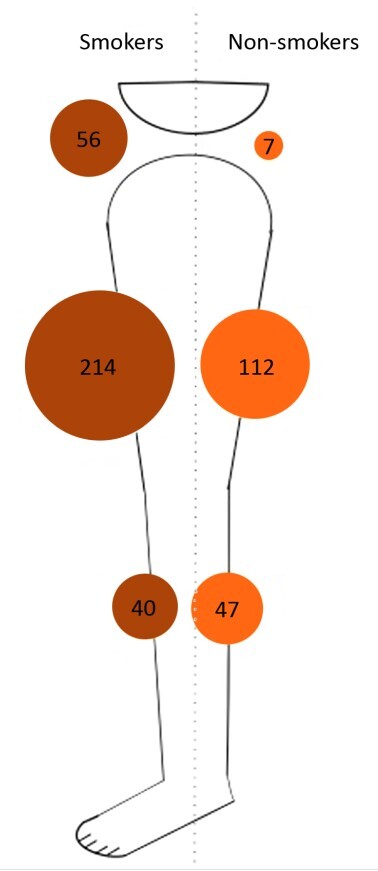
Ilustration of the anatomic distribution of peripheral arterial disease (aortoiliac, femoropopliteal, or infrapopliteal) in smokers and non-smokers.

The univariate analysis of the risk factors for atherosclerosis in relation to the anatomic distribution of PAD showed that patients of both sexes, diabetics, and smokers exhibited significant differences in the anatomic distribution of proximal PAD obstruction (Table 2). In the multivariate analysis, the three different PAD distributions were analyzed as binomial variables (Table 3). In the multivariate analysis, women exhibited a 2.7 (CI: 1.75-4.26/ p = 0.00001) times greater chance of femoropopliteal involvement. Smokers had a 3.6 (CI: 1.54-8.30/ p = 0.003) times greater risk of aortoiliac disease. Diabetics exhibited a 1.8 (CI: 1.04-3.19/ p = 0.03) times greater chance of only infrapopliteal occlusive disease, with pervious arteries in the aortoiliac and femoropopliteal regions. Neither arterial hypertension or age were statistically significant in univariate or multivariate analyses. Figures 2, 3, and 4 illustrate the distribution of atherosclerosis risk factors that were significant in the multivariate analysis and the rates of proximal PAD obstruction at the three different anatomic levels (aortoiliac, femoropopliteal, and infrapopliteal). In these figures, it can be observed that femoropopliteal obstruction is the most common level in all groups of patients, regardless of risk factors. These figures also illustrate the predominance of women among femoropopliteal PAD patients (Figure 2), the low risk of isolated infrapopliteal disease in non-diabetic patients (Figure 3), and the impact of smoking on obstruction at the aortoiliac level, with a low number of non-smokers with proximal obstruction at this level (Figure 4).

**Table 2:** Univariate analysis of associations between risk factors for atherosclerosis and the anatomic distribution of peripheral arterial disease.

Risk factors for atherosclerosis (N=476)	Anatomic distribution of PADa			p*
	Aortoiliac (63)	Femoropopliteal (326)	Infrapopliteal (87)	
	N(%)	N(%)	N(%)	
Age (mean/years)	67.1 (±8.7)	69.5 (±10.5)	68.0(±9.9)	0.16
Gender				
Men	47 (19%)	150 (60%)	52 (21%)	0.0001
Women	16 (7%)	176 (78%)	35 (15%)	
Diabetes mellitus				
Diabetic	26 (9%)	185 (68%)	62 (23%)	0.001
Not diabetic	37 (18%)	141 (70%)	25 (12%)	
Arterial hypertension				
Hypertensive	45 (12%)	251 (69%)	71 (19%)	0.3
Not hypertensive	18 (16%)	75 (69%)	16 (15%)	
Smoking				
Smokers	56 (18%)	214 (69%)	40 (13%)	0.00001
Non-smokers	07 (4%)	112 (68%)	47 (28%)	

* Chi-square (categorical variables) and analysis of variance ANOVA (continuous variables).

ªPeripheral arterial disease.

**Table 3:** Multivariate logistic regression analysis of associations between risk factors for atherosclerosis and the anatomic distribution^b^ of peripheral arterial disease.

Anatomic distribution of PADª (N=476)	*Odds Ratio*	Confidence interval	p	
	N(%)	N(%)		
Aortoiliac PADª				
*Age*	0.99	0.96-1.02	0.38	
*Gender (men)*	2.23	1.18-4.23	0.01	
*Diabetes mellitus*	0.69	0.38-1.23	0.21	
*Arterial hypertension*	1.25	0.65-2.43	0.5	
*Smoking*	3.57	1.54-8.30	0.003	
Femoropopliteal PADª			
*Age*	1.02	0.99-1.04	0.07	
*Gender (Women)*	2.73	1.75-4.26	0.00001	
*Diabetes mellitus*	0.84	0.54-1.30	0.44	
*Arterial hypertension*	0.74	0.44-1.24	0.25	
*Smoking*	1.43	0.91-2.26	0.12	
Infrapopliteal PADª				
*Age*	0.98	0.96-1.01	0.17	
*Sex (men)*	2.43	1.41-4.17	0.001	
*Diabetes mellitus*	1.83	1.04-3.19	0.03	
*Arterial hypertension*	1.31	0.67-2.55	0.43	
*Smoking*	0.32	0.19-0.55	0.0001	

ªPeripheral arterial disease.

b Anatomic distribution of proximal obstruction diagnosed by physical examination (pulses) and confirmed with imaging methods (duplex scan, angiotomography, and/or digital subtraction angiography).

## DISCUSSION

This study found that risk factors for atherosclerosis influenced the distribution of atherosclerotic obstructive disease of the lower limbs in patients with chronic critical lower limb ischemia. The femoropopliteal region was the most often involved in patients with advanced PAD and CLTI. The multivariate analysis showed that patients with diabetes had a higher likelihood of having PAD that only involved the infrapopliteal region, with patent aortoiliac and femoropopliteal segments. There was a predominance of women among patients with femoropopliteal involvement and smoking conferred a higher chance of aortoiliac PAD. Arterial hypertension did not affect the pattern of anatomic distribution of proximal PAD obstruction in patients with CLTI.

Other studies have also found that risk factors for atherosclerosis influence the anatomic distribution of PAD.^16,18,19^ As in this study, other authors also observed that female sex was associated with femoropopliteal disease and that smoking was associated with more proximal PAD involvement at the aortoiliac level.^18^ Diehm et al.^18^ analyzed the atherosclerosis risk factors age, sex, DM, SAH, and smoking in relation to different PAD locations among patients scheduled for angioplasty.^18^ However, in contrast with our study, Diehm et al.^18^ found that age influenced PAD pattern, with younger patients more frequently exhibiting iliac disease.^18^ However, these authors included patients with claudication in their sample, whereas the present study only enrolled patients with CLTI, the majority of whom had tissue loss/gangrene.

In this study, almost half (48%) of the population of patients with advanced PAD and CLTI were women, who exhibited a stronger association with PAD in the femoropopliteal region, similar to what has been observed by other authors.^18^ Global statistics have shown that women have higher rates of both prevalence and incidence of PAD after 40 years of age.^3^ Studies suggest sex differences in some biomarkers of atherosclerosis, identifying C-reactive protein (CRP) as a factor related to PAD in women.^20^ In Brazil, a cross-sectional study that enrolled more than 1,000 participants over the age of 30 found higher CRP concentrations among women.^21^ The prevalence of risk factors for atherosclerosis also differs by sex. In the Brazilian population, the estimated prevalence of DM is higher among women (10.2%) than men (8.1%), and there are also differences between different regions of the country.^10^ In the Northeast, the prevalence of DM among women was 14.7%, higher than the prevalence among Brazilian men, which is 8.1%.^10^ Diabetes may be one of the reasons why more women than men have been affected by PAD, with higher incidence and prevalence rates.^3^

In contrast with smoking, which is in decline, DM is one of the risk factors for PAD that is increasing in exposure worldwide.^6^ Globally, the incidence of DM increased by 102.9% from 1990 to 2017 (from 11.3 million to 22.9 million people).^8^ The increase in exposure to metabolic risk factors such as obesity and DM is a challenge in the fight against CVD and, consequently, against PAD, all over the world.^6^ In Brazil, female sex, age, low educational level, overweight, and obesity are all factors associated with higher DM prevalence.^22^ A systematic review of the literature reported a difference in the anatomic distribution of PAD, with a significantly lower frequency of aortoiliac involvement among diabetics and higher rates of infrapopliteal involvement.^16^ However, the review highlighted the use of different definitions of significant disease/obstruction and the variety of different scales and levels used in analyses of the anatomic distribution of PAD.^16^ The present study analyzed proximal occlusions with clinical repercussions, absence of pulses, and CLTI, finding that both diabetics and non-diabetics with CLTI most often had proximal obstruction at the femoropopliteal level - whereas diabetics also had a higher chance of PAD involving only the infrapopliteal arteries. One possible interpretation of these results is that diabetics have only infrapatellar involvement more frequently than non-diabetics, maintaining patency of the aortoiliac and femoropopliteal segments. In Brazil, a study of angiographic findings in PAD found that diabetic patients exhibited a predominance of lesions classified as TASC A and B in the femoropopliteal zone, whereas non-diabetics had a predominance of TASC C and D classifications, indicating more advanced injuries.^23^ Histologically, a study showed that the pattern of atherosclerosis in the arteries of the leg is similar in diabetics and non-diabetics.^24^ Other authors studied type 2 DM patients with symptomatic PAD, finding an influence from glycated hemoglobin (HbA1c) on the pattern of the anatomic distribution of PAD.^19^ In these authors’ results, patients with HbA1C exceeding 7.5 more frequently exhibited multisegmental PAD and involvement of the femoropopliteal and crural segments, in a sample that also included claudicant patients.^19^

Smoking is an important risk factor for CVD and PAD.^12,14^ In agreement with the literature, smoking was associated with an increased risk of aortoiliac involvement in this study.^16,18^ Other studies have identified a positive association between smoking and calcification of the aortoiliac segment, considering it a risk factor for aortic calcification.^25,26^ A longitudinal cohort study demonstrated an association between smoking and increased incidence of the three major CVDs, in particular PAD, finding that smokers with a habit lasting 35 years or more had a 5.56 times greater risk of PAD, a 2.30 times greater risk of coronary disease, and 1.92 times greater risk of stroke than people who had never smoked.^27^ Even though the incidence of PAD declines 1 year after quitting smoking, the study showed that the risk of PAD is only similar to that for people who had never smoked after 30 years of abstinence, while the same was true for coronary disease after 20 years.^27^

This study has limitations. It was an observational, cross-sectional, and retrospective study that only enrolled patients with CLTI and defined the anatomic distribution of PAD according to physical examination of pulses, confirmed with imaging exams showing the pattern of proximal obstruction causing the absence of pulses. As such, the study illustrates the pattern of proximal PAD with obstruction and absence of pulses and did not analyze possible stenosis or irregularities of the wall in the entire arterial tree. An additional limitation is that, because of the retrospective design, other risk factors for atherosclerosis, such as hypercholesterolemia or family history, and details about smoking were not analyzed. Information about ethnicity was also unavailable in the medical records. Nevertheless, the sample included patients hospitalized for CLTI and the majority of patients enrolled had already been given clinical treatment with statins prior to admission for advanced PAD. Among these patients with advanced PAD and CLTI, our study attempted to trace the impact of risk factors such as sex, age, smoking, and DM on the pattern of anatomic distribution of chronic atherosclerotic obstructions. Atherosclerosis is the principal cause of CVD and has been studied as a chronic inflammatory disease in which multiple risk factors interact with the artery wall in a complex manner.^28^ The literature suggests that complex molecular mechanisms interact in the formation and distribution of different atherosclerotic lesions in different parts of the body,^18^ which shows the importance of studying the epidemiology of PAD in each site and in different countries.

## CONCLUSIONS

Concluding, the atherosclerosis risk factors gender, DM, and smoking influence the pattern of anatomic distribution of PAD in patients with chronic limb-threatening ischemia. Femoropopliteal is the most common occlusive level in all groups of patients, regardless of risk factors. Diabetic patients had a higher chance of their PAD occlusions involving only the infrapopliteal arteries. Women had a higher chance of obstruction of the femoropopliteal segment. Smokers had a higher risk of obstructive PAD involving the aortoiliac region.
