# Supplementary material for: Influence of atherosclerosis risk factors on the anatomical distribution of peripheral arterial disease in patients with chronic limb-threatening ischemia: a cross-sectional study
Source: J Vasc Bras. 2023 Jul 17;22:e20230014. doi: 10.1590/1677-5449.202300141 (PMC10421571; doi:10.1590/1677-5449.202300141)
Supplement: Translation

# Translation: Influência dos fatores de risco para aterosclerose na distribuição anatômica da doença arterial periférica em pacientes com isquemia crônica crítica de membros: um estudo transversal

**DOI:** 10.1590/1677-5449.202300142

**Authors:** Vanessa Prado dos Santos, Camila Izabel Cerutti, Marcelo José Carlos Alencar, André Brito Queiroz, Lucas de Mello Ferreira, Cícero Fidelis, José Siqueira de Araújo, Carlos Alberto Silveira Alves

**Affiliations:** 1 Universidade Federal da Bahia - UFBA, Hospital Universitário Professor Edgard Santos - HUPES, Salvador, BA, Brasil.

**Keywords:** aterosclerose, isquemia crônica crítica de membros, doença arterial periférica, diabetes *mellitus*, fatores de risco

## Abstract

**Contexto:**

Os fatores de risco para aterosclerose podem ter influência distinta nas doenças cardiovasculares, impactando também no território da doença arterial periférica (DAP).

**Objetivos:**

Estudar a influência dos diferentes fatores de risco para a aterosclerose na distribuição anatômica da DAP em pacientes com isquemia crítica crônica de membros (ICCM).

**Métodos:**

Foi realizado um estudo observacional, transversal e analítico, incluindo 476 doentes internados devido à ICCM por DAP. Foram estudados, comparativamente, os pacientes com DAP dos três distintos territórios anatômicos (aortoilíaco, femoropoplíteo e infragenicular) em relação à presença dos fatores de risco para a aterosclerose (idade, gênero, diabetes *mellitus*, tabagismo e hipertensão arterial). A análise multivariada foi realizada para testar a associação entre os fatores de risco e a distribuição anatômica da DAP.

**Resultados:**

A média de idade dos pacientes foi de 69 anos, 249 (52%) eram homens, e 273 (57%), diabéticos. Predominaram os pacientes na Categoria 5 da Classificação de Rutherford (353/74%). Na análise multivariada, as mulheres tiveram chance 2,7 (IC: 1,75-4,26) vezes maior de doença do território femoropoplíteo. Os doentes tabagistas tiveram 3,6 (IC: 1,54-8,30) vezes maior risco de doença do território aortoilíaco. Diabéticos apresentaram 1,8 (IC: 1,04-3,19) vezes maior chance de obstrução apenas do território infragenicular.

**Conclusões:**

O estudo mostrou que gênero, diabetes *mellitus* e tabagismo influenciam no padrão de distribuição da DAP em pacientes com ICCM. Os doentes diabéticos apresentaram maior chance de doença isolada do território infragenicular, as mulheres tiveram maior risco de DAP do território femoropoplíteo e os tabagistas possuem maior chance comprometimento aortoilíaco.

## INTRODUÇÃO

As doenças cardiovasculares (DCVs) são a principal causa de mortalidade no Brasil e no mundo^1^ . A aterosclerose é a maior causa de DCV, e a doença cardíaca isquêmica, o acidente vascular cerebral (AVC) e a doença arterial periférica (DAP) são os três principais diagnósticos relacionados à doença^2^ . Globalmente, em números absolutos, houve um aumento de 71,5% nos casos novos de DAP, entre 1990 e 2019, chegando a 10.504.092 casos em 2019^3^ . No entanto, quando se considera a prevalência de DAP ajustada por idade, houve uma queda de 21,7% entre 1990 e 2019, principalmente em países de renda e índice sociodemográfico elevado^3^ . No Reino Unido, a incidência e a prevalência de DAP sintomática apresentaram um declínio entre 2000 e 2014, com um decréscimo de prevalência de 3,4 para 2,4%^4^ . O Brasil faz parte de um grupo de países com incidência estimada de DAP, ajustada por idade, abaixo de 100 pessoas por 100 mil habitantes^3^ . A prevalência de DAP aumenta com a idade em ambos os sexos, sendo a maior incidência na faixa etária de 90-94 anos para os homens, e 75-79 anos para as mulheres^3^ . De maneira geral, as taxas de prevalência e de incidência de DAP, após os 40 anos de idade, têm sido maiores entre as mulheres que entre os homens^3^ .

Idade avançada, diabetes *mellitus* (DM), hipertensão arterial sistêmica (HAS) e tabagismo são fatores comumente associados ao risco de DCV e DAP, que têm na aterosclerose sua principal etiologia^5^ . Considerando o comportamento dos fatores de risco para DAP, entre 2010 e 2019 houve uma redução na exposição ao tabagismo e um aumento, maior que 1% por ano, na exposição à hiperglicemia no mundo todo^6^ . Globalmente, o risco de exposição à hiperglicemia apresentou um aumento significativo de 1,32% (1,01 a 1,64) por ano, enquanto o tabagismo declinou em 1,20% (-1,29 a -1,11)^6^ . Quando comparada à hiperglicemia, a hipertensão arterial apresentou um menor aumento na exposição, de 0,51% (0,04 a 1,00) por ano, entre 2010 e 2019^6^ . A prevalência de HAS na população adulta no Brasil, considerando a medida por instrumento e/ou em uso de medicação anti-hipertensiva, é de 32,3%, sendo que a prevalência aumenta com a idade, chegando a 71,7% na faixa etária acima de 70 anos^7^ .

O DM representa um grave problema de saúde pública no mundo todo^8^ . Ao longo dos anos, a prevalência de DM vem crescendo em diferentes países^9,10^ . Nos EUA, a prevalência estimada no período compreendido entre 1999 e 2002 era de 9,5%, chegando a 12% entre 2013 e 2016^9^ . Em 2018, a estimativa era de mais de 34 milhões de pessoas com DM, em uma prevalência estimada de 13% da população adulta, chegando a 26,6% entre pessoas com 65 anos ou mais^9^ . No Brasil, a prevalência estimada de DM é de 9,2%, com uma marcada diferença entre as diferentes regiões, sendo de 6,3% na região Norte, 7,2% no Sul, 7,6% no Centro-Oeste, 12,2% no Nordeste e 12,8% na região Sudeste^10^ .

Diabetes e DAP contribuem para um maior risco de amputações dos membros inferiores em pacientes com úlceras e lesões necróticas^11,12^ . Na DAP, os doentes portadores de isquemia crônica crítica de membros (ICCM) ou *Critical Limb-threatening Isquemia* (CLTI) são aqueles que cursam com dor ao repouso, gangrena ou úlcera de membro inferior com duração maior que 2 semanas, apresentando doença avançada e um maior risco de perda de membro^12,13^ . Estima-se que 1 a 3% dos portadores de DAP tenham como apresentação clínica inicial os sintomas de isquemia crítica de membro; contudo, a epidemiologia da ICCM não está claramente estabelecida^12,13^ . Nos EUA, enquanto a prevalência estimada de DAP foi de 10,69%, a prevalência de isquemia crítica de membro foi de 1,33%, sendo maior entre diabéticos e entre doentes que tiveram AVC ou falência cardíaca^14^ . Também nos EUA, mais de 130.000 hospitalizações em doentes diabéticos, no ano de 2016, estiveram relacionadas às amputações^9^ . No mundo, em 2016, havia cerca de 6,8 milhões de pessoas que sofreram amputações relacionadas à complicações do DM^11^ .

Considerando a prevalência e a morbidade da DAP, da ICCM e dos seus fatores de risco, o objetivo deste estudo foi identificar a influência dos diferentes fatores de risco ateroscleróticos na distribuição anatômica da DAP em pacientes com isquemia crônica crítica de membros inferiores. A hipótese do estudo é que os diferentes fatores de risco ateroscleróticos influenciam na distribuição anatômica do nível de obstrução proximal da DAP.

## MÉTODOS

Foi realizado um estudo observacional, transversal e retrospectivo, incluindo consecutivamente os pacientes internados com o diagnóstico de isquemia crônica crítica de membro inferior secundária à DAP, no Hospital Universitário Professor Edgard Santos (HUPES) da Universidade Federal da Bahia (UFBA), entre 2014 e 2017. Tratando-se de um estudo observacional, foram utilizadas as recomendações da iniciativa *Strengthening the Reporting of Observational Studies in Epidemiology* ^15^ para sua descrição. Foram incluídos consecutivamente 476 pacientes internados para o tratamento de ICCM secundária à DAP de etiologia aterosclerótica. O cálculo do tamanho da amostra considerou um erro alfa de 0,05, um poder de estudo de 80% e uma revisão sistemática da literatura^16^ sobre a proporção de DAP dos três diferentes territórios anatômicos (aortoilíaco, femoropoplíteo e tibial/infragenicular) em pessoas com e sem DM. O cálculo resultou em um tamanho mínimo de amostra de 114 pacientes quando se considerou a diferença entre pessoas com e sem DM e doença do território aortoilíaco, 408 casos para o território femoropoplíteo e 242 doentes para o território infra-poplíteo. O fluxograma de inclusão dos casos se encontra na Figura 1. O projeto de pesquisa foi aprovado pelo Comitê de Ética em Pesquisa da mesma instituição, com número do parecer 2.441.556.

**Figura 1:** Fluxograma de seleção, exclusão e inclusão dos casos analisados.

A coleta de dados foi realizada através da revisão de prontuários, fichas de acompanhamento clínico e exames de imagem arquivados de pacientes internados com diagnóstico de ICCM no Serviço de Cirurgia Vascular do Hospital Universitário Professor Edgard Santos (HUPES). Todos os pacientes incluídos apresentavam dor ao repouso, gangrena (com perda tecidual maior ou menor) ou úlcera com duração superior a 2 meses. Foram estudados os fatores de risco para aterosclerose (sexo/gênero, idade, HAS, DM e tabagismo) e sua possível associação com o território anatômico da DAP (aortoilíaco, femoropoplíteo ou infragenicular) encontrado no exame físico e confirmado por método de imagem. Os pacientes foram divididos em três grupos, através do exame físico dos pulsos e de exame de imagem, conforme a distribuição anatômica ou território da DAP (aortoilíaco, femoropoplíteo e infragenicular), indicando o local de obstrução proximal da árvore arterial. Os pacientes sem pulso femoral no membro foram considerados portadores de DAP do território aortoilíaco. Os doentes com pulso femoral normal e sem pulso poplíteo no membro acometido foram considerados com doença femoro-poplítea. Os casos que apresentavam pulsos femorais e poplíteos normais e ausência dos pulsos pedioso e tibial posterior foram considerados infrageniculares. A ausência de pulsos dos casos incluídos atestava que havia oclusão arterial crônica ou estenose crítica do segmento arterial proximal, sendo confirmado por exame de imagem. Todos os pacientes incluídos no estudo cursavam com ausência de ambos os pulsos ao nível do pé (pedioso e tibial posterior) no exame físico. Todos os doentes realizaram método de imagem que comprovava a doença arterial obstrutiva periférica e o seu território (*duplex scan*, angiotomografia e/ou angiografia de subtração digital). Na revisão dos prontuários e dos métodos de imagem dos casos incluídos pode-se verificar a descrição da presença de fluxo ou opacificação das três artérias da perna (tibial anterior, tibial posterior e fibular) em apenas 30 doentes da amostra (6,3%), revelando que a maioria apresentava doença multissegmentar, com comprometimento das artérias infrageniculares. Apesar do índice tornozelo-braquial ser rotineiramente calculado no serviço, o dado não foi analisado no estudo.

Os 3 grupos de pacientes, de acordo com distribuição da obstrução proximal da DAP, foram estudados comparativamente quanto à presença dos fatores de risco para aterosclerose (idade, gênero, DM, HAS e tabagismo). Foram considerados diabéticos aqueles que já tinham diagnóstico prévio da doença e se encontravam em tratamento da mesma. Esse também foi o critério utilizado para a HAS. O antecedente de cardiopatia e de doença renal crônica foi considerado de acordo com o registro em prontuário do diagnóstico, por meio de história clínica do paciente e do registro dos profissionais assistentes. Considerando o desenho retrospectivo do estudo, através da revisão de prontuários, o tabagismo foi considerado quando havia o registro de que a pessoa era tabagista quando admitido na unidade hospitalar.

Os dados da pesquisa foram verificados por meio do programa Epi-Info^TM^, versão 7.2.2.6. A análise descritiva foi realizada para as variáveis categóricas e contínuas. Para estudar a associação das variáveis categóricas (os fatores de risco gênero, DM, HAS e tabagismo) e a distribuição anatômica da DAP (aortoilíaca, femoro-poplítea ou infragenicular), foi realizada a análise univariada através do teste do qui-quadrado. As variáveis contínuas, como idade, tiveram suas médias estudadas comparadas pela análise de variância (ANOVA). Foi realizada análise multivariada, através da regressão logística, para testar a possível associação entre os cinco fatores de risco ateroscleróticos e o padrão de distribuição anatômica da DAP. Na análise multivariada, a distribuição anatômica da DAP (nível de obstrução proximal) foi analisada como variável binomial (aortoilíaca *versus* não aortoilíaca; femoro-poplítea *versus* não femoro-poplítea; e infragenicular *versus* não infragenicular). Adotou-se o nível de significância de 5% (p < 0,05) para rejeição da hipótese nula.

## RESULTADOS

Quatrocentos e setenta e seis (476) pacientes foram incluídos no estudo. A média de idade dos doentes foi de 69 (±10 anos); 249 (52%) eram homens, 273 (57%) eram diabéticos, 367 (77%) hipertensos e 310 (65%) tabagistas. Quanto à distribuição anatômica da obstrução proximal da DAP, através do exame dos pulsos dos membros inferiores confirmado por método de imagem, 63 (13%) dos doentes apresentavam obstrução proximal no território aortoilíaco, 326 (69%), oclusão femoro-poplítea, com ausência de pulsos poplíteos e distais ao exame físico, e os demais 87 (18%) tinham acometimento do território infragenicular, apresentando pulso poplíteo normal e pulsos distais ausentes. O nível de obstrução proximal foi confirmado por método de imagem, e nenhum doente incluído na amostra possuía pulsos presentes no nível do tornozelo. Quanto à Classificação de Rutherford^17^ para isquemia crônica, 353 (74%) dos membros estavam na categoria 5 e 81, (17%) na categoria 6, e confirmando a presença de perda tecidual 434 (91%) da amostra. O estudo descritivo das características da amostra se encontra detalhado na Tabela 1.

**Tabela 1:** Características da amostra (476 doentes) incluída com doença arterial periférica e isquemia crítica de membro inferior.

Características da amostra (N = 476)		N (%)
Homens		249 (52%)
Idade (média/anos)		69 (±10) anos
Tabagismo		310 (65%)
Diabetes *mellitus*		273 (57%)
Hipertensão arterial		367 (77%)
Antecedente de cardiopatia		103 (22%)
Doença renal crônica		34 (7%)
Tempo de duração da lesão trófica (média/dias)		98 (±76) dias
Classificação de Rutherford	Categoria 4	42 (9%)
	Categoria 5	353 (74%)
	Categoria 6	81 (17%)
Distribuição anatômica da DAP^a^	Aortoilíaca	63 (13%)
	Femoro-poplítea	326 (69%)
	Infragenicular	87 (18%)

a Doença arterial periférica.

A análise univariada dos fatores de risco para aterosclerose, em relação à distribuição anatômica da DAP, mostrou que os pacientes de ambos os gêneros, diabéticos e tabagistas apresentaram diferença significativa na distribuição anatômica da obstrução proximal por DAP (Tabela 2). Na análise multivariada, os 3 territórios da DAP foram analisados como variáveis binomiais (Tabela 3). Na análise multivariada, as mulheres apresentaram uma chance 2,7 (IC: 1,75-4,26/ p = 0,00001) vezes maior de acometimento do território femoropoplíteo. Os doentes tabagistas tiveram 3,6 (IC: 1,54-8,30/ p = 0,003) vezes maior risco de doença do território aortoilíaco. Os doentes diabéticos apresentaram 1,8 (IC: 1,04-3,19/ p = 0,03) vezes maior chance de obstrução apenas do território infragenicular, com perviedade das artérias do setor aortoilíaco e do femoropoplíteo. Hipertensão arterial e idade não apresentaram significância estatística nas análises uni ou multivariada. A visualização dos fatores de risco ateroscleróticos, significantes na análise multivariada, e a presença de obstrução proximal por DAP dos três diferentes territórios anatômicos (aortoilíaco, femoropoplíteo e infragenicular) se encontram nas Figuras 2, 3 e 4. Nas figuras, é possível observar que a obstrução femoro-poplítea é topografia mais frequente de DAP em todos os grupos de doentes e seus fatores de risco. A visualização também evidencia o predomínio das mulheres entre os doentes da DAP femoro-poplítea (Figura 2), o baixo risco de doença infragenicular isolada em pacientes não diabéticos (Figura 3) e o impacto do tabagismo na obstrução do território aortoilíaco, com um número reduzido de não tabagistas com o nível de obstrução proximal nesse território (Figura 4).

**Tabela 2:** Análise univariada da associação entre os fatores de risco ateroscleróticos e a distribuição anatômica da doença arterial periférica.

Fatores de risco para aterosclerose (N=476)	Distribuição anatômica da DAPa			Valor de p*
	Aortoilíaca (63)	Femoro-poplítea (326)	Infragenicular (87)	
	N(%)	N(%)	N(%)	
Idade (*média/anos*)	67,1 (±8,7)	69,5 (±10,5)	68,0(±9,9)	0,16
Gênero				
*Homens*	47 (19%)	150 (60%)	52 (21%)	0,0001
*Mulheres*	16 (7%)	176 (78%)	35 (15%)	
Diabetes *mellitus*				
*Diabéticos*	26 (9%)	185 (68%)	62 (23%)	0,001
*Não diabéticos*	37 (18%)	141 (70%)	25 (12%)	
Hipertensão arterial				
*Hipertensos*	45 (12%)	251 (69%)	71 (19%)	0,3
*Não hipertensos*	18 (16%)	75 (69%)	16 (15%)	
Tabagismo				
*Tabagistas*	56 (18%)	214 (69%)	40 (13%)	0,00001
*Não tabagistas*	07 (4%)	112 (68%)	47 (28%)	

* Qui-quadrado (variáveis categóricas) e análise de variância ANOVA (variáveis contínuas).

ª Doença arterial periférica.

**Tabela 3:** Análise multivariada através da regressão logística da associação entre os fatores de risco ateroscleróticos e a distribuição anatômica^b^ da doença arterial periférica.

Distribuição anatômica da DAPª (N=476)	*Odds Ratio*	Intervalo de Confiança	Valor de p	
	N(%)	N(%)		
DAPª do território aortoilíaco				
*Idade*	0,99	0,96-1,02	0,38	
*Gênero (homens)*	2,23	1,18-4,23	0,01	
*Diabetes* mellitus	0,69	0,38-1,23	0,21	
*Hipertensão arterial*	1,25	0,65-2,43	0,5	
*Tabagismo*	3,57	1,54-8,30	0,003	
DAPª do território femoropoplíteo			
*Idade*	1,02	0,99-1,04	0,07	
*Gênero (Mulheres)*	2,73	1,75-4,26	0,00001	
*Diabetes* mellitus	0,84	0,54-1,30	0,44	
*Hipertensão arterial*	0,74	0,44-1,24	0,25	
*Tabagismo*	1,43	0,91-2,26	0,12	
DAPª do território infragenicular				
*Idade*	0,98	0,96-1,01	0,17	
*Gênero (homens)*	2,43	1,41-4,17	0,001	
*Diabetes* mellitus	1,83	1,04-3,19	0,03	
*Hipertensão arterial*	1,31	0,67-2,55	0,43	
*Tabagismo*	0,32	0,19-0,55	0,0001	

ªDoença arterial periférica.

b Distribuição anatômica da obstrução proximal diagnosticada através do exame físico (pulsos) e confirmada por método de imagem (*duplex scan*, angiotomografia e/ou angiografia de subtração digital).

**Figura 2:** Representação gráfica da distribuição anatômica da doença arterial periférica (aortoilíaca, femoropoplítea e infragenicular) entre os diferentes gêneros (homens e mulheres).

**Figura 3:** Representação gráfica da distribuição anatômica da doença arterial periférica (aortoilíaca, femoropoplítea e infragenicular) entre os portadores de diabetes *mellitus* (DM) e não portadores de DM.

**Figura 4:** Representação gráfica da distribuição anatômica da doença arterial periférica (aortoilíaca, femoropoplítea e infragenicular) entre tabagistas e não tabagistas.

## DISCUSSÃO

O presente estudo constatou que os fatores de risco para aterosclerose influenciam na distribuição da doença obstrutiva aterosclerótica dos membros inferiores em pacientes com isquemia crônica crítica de membro inferior. Considerando os doentes com DAP avançada e ICCM, o território femoropoplíteo é o mais frequentemente acometido. A análise multivariada mostrou que doentes diabéticos apresentam maior chance de que a DAP acometa apenas o território infragenicular, com perviedade dos segmentos aortoilíaco e femoropoplíteo. As mulheres apresentam predominância no comprometimento do território femoropoplíteo, e o tabagismo implica em uma maior chance de DAP do território aortoilíaco. A hipertensão arterial não influenciou no padrão de distribuição anatômica da obstrução proximal da DAP em doentes com ICCM.

Outros estudos também identificaram que fatores de risco ateroscleróticos influenciam na distribuição anatômica da DAP^16,18,19^ . Assim como neste estudo, outros autores também verificaram que o gênero feminino esteve associado à doença femoro-poplítea, e o tabagismo, ao acometimento mais proximal da DAP do setor aortoilíaco^18^ . Diehm et al.^18^ analisaram os fatores de risco ateroscleróticos idade, gênero, DM, HAS e tabagismo em relação aos diferentes territórios da DAP, entre doentes que seriam tratados por angioplastia^18^ . Contudo, diferentemente do nosso estudo, Diehm et al.^18^ encontraram influência da idade no padrão de DAP, com os mais jovens apresentando mais frequentemente a doença do território ilíaco^18^ . No entanto, os autores incluíram pacientes claudicadores na amostra, enquanto o estudo aqui apresentado contou apenas com doentes portadores de ICCM, sendo a maioria com perda tecidual/gangrena.

Neste estudo, quase metade (48%) da população de pacientes com DAP avançada e ICCM foi composta por mulheres, que apresentaram maior associação com DAP do território femoropoplíteo, semelhante ao achado de outros autores^18^ . Estatística globais têm revelado que as mulheres apresentam maiores taxas de prevalência e de incidência de DAP após os 40 anos de idade^3^ . Estudos sugerem diferenças em alguns biomarcadores para aterosclerose nos distintos gêneros, apontando a proteína C reativa (PCR) como um fator relacionado à DAP em mulheres^20^ . No Brasil, estudo transversal que incluiu mais de 1.000 participantes acima dos 30 anos encontrou maiores concentrações de PCR entre mulheres^21^ . Os fatores de risco para aterosclerose também têm prevalência diferente entre os gêneros. Na população brasileira, a prevalência estimada de DM é maior entre as mulheres (10,2%) do que entre os homens (8,1%), havendo também diferenças entre as regiões do país^10^ . Na região Nordeste, a prevalência de DM entre as mulheres foi de 14,7%, acima da prevalência entre os homens brasileiros, que é de 8,1%^10^ . O DM pode ser uma das possíveis explicações para que, globalmente, mais mulheres do que homens tenham sido afetadas pela DAP, com maior taxa de incidência e prevalência^3^ .

Entre os fatores de risco para DAP, diferentemente do tabagismo, que se encontra em declínio, o DM vem demonstrando aumento da exposição no mundo todo^6^ . Globalmente, a incidência de DM apresentou um aumento de 102,9% entre 1990 e 2017 (11,3 milhões para 22,9 milhões de pessoas)^8^ . O aumento na exposição aos fatores de risco metabólicos, como obesidade e DM, é um desafio no combate às DCVs e, consequentemente, à DAP, no mundo todo^6^ . No Brasil, sexo feminino, idade, baixa escolaridade, sobrepeso e obesidade são fatores associados à maior prevalência de DM^22^ . Revisão sistemática da literatura apontou uma diferença na distribuição anatômica da DAP, com frequência significativamente menor do território aortoilíaco entre diabéticos e maior de comprometimento do território infragenicular^16^ . No entanto, a revisão chama a atenção para a utilização de diferentes definições sobre doença/obstrução significante e para a variedade de escalas e territórios considerados na análise da distribuição anatômica da DAP^16^ . O estudo aqui apresentado analisou oclusões proximais com repercussão clínica, ausência de pulsos e ICCM, verificando que tanto diabéticos como não diabéticos com ICCM tiveram mais comumente a obstrução proximal no setor femoropoplíteo - sendo que os diabéticos apresentaram maior chance de que a DAP acometesse apenas o território infragenicular. A possível interpretação desses resultados é que os diabéticos, mais frequentemente que os não diabéticos, apresentam comprometimento apenas do território infrapatelar, mantendo a perviedade dos territórios aortoilíaco e femoropoplíteo. No Brasil, um estudo sobre as alterações angiográficas na DAP constatou que doentes diabéticos apresentaram predomínio das lesões nas classificações TASC A e B no território femoropoplíteo, enquanto os não diabéticos tiveram predomínio das classificações TASC C e D, denotando lesões mais avançadas^23^ . Histologicamente, uma pesquisa revelou que o padrão de aterosclerose das artérias da perna é semelhante em diabéticos e não diabéticos^24^ . Outros autores, estudando portadores de DM do tipo 2 com DAP sintomática, encontraram influência da hemoglobina glicada (HbA1c) no padrão de distribuição anatômica da DAP^19^ . Nos resultados dos autores, os doentes com HbA1C acima de 7,5 apresentaram maior ocorrência de DAP multissegmentar e de comprometimento do segmento femoropoplíteo e crural, sendo que a amostra também contou com pacientes claudicadores^19^ .

O tabagismo é um importante fator de risco para DCV e DAP^12,14^ . Concordando com a literatura, o tabagismo esteve associado a um maior risco de comprometimento do território aortoilíaco neste estudo^16,18^ . Outros estudos apontam uma associação positiva entre o tabagismo e a presença de calcificação no território aortoilíaco, considerando-o um fator de risco para a calcificação aórtica^25,26^ . Pesquisa de coorte longitudinal demonstrou a associação do tabagismo com uma maior incidência das três principais DCVs, com destaque para a DAP, sendo que tabagistas com duração do hábito maior ou igual a 35 anos apresentaram um risco 5,56 vezes maior de DAP, 2,30 maior de doença coronariana e 1,92 de AVC quando comparados a quem nunca fumou^27^ . Mesmo havendo queda na incidência de DAP após um ano de cessação do tabagismo, o estudo mostrou que o risco de DAP só se assemelha ao daqueles que nunca fumaram após 30 anos de manutenção da cessação do tabagismo, o mesmo ocorrendo após 20 anos quando se trata da doença coronariana^27^ .

O estudo realizado apresentou limitações. Trata-se de um estudo observacional, transversal e retrospectivo, que incluiu apenas pacientes com ICCM, e considerou a distribuição anatômica da DAP através do exame físico de pulsos, confirmada através de exames de imagem, mostrando o padrão de obstrução proximal, que determina a ausência de pulsos. Sendo assim, a pesquisa revelou o padrão de DAP proximal com obstrução e ausência de pulsos, não analisando possíveis estenoses ou irregularidades parietais em toda a árvore arterial. Uma limitação adicional é que, devido ao seu desenho retrospectivo, outros fatores de risco para aterosclerose, como hipercolesterolemia ou história familiar, e detalhes sobre o tabagismo não foram analisados. Informações sobre a etnia também não estavam disponíveis nos prontuários. Contudo, a amostra considerou doentes hospitalizados por ICCM, e a maioria dos pacientes incluídos já recebia tratamento clínico com estatinas anteriormente à internação devido à DAP avançada. Entre pacientes portadores de DAP avançada e ICCM, o nosso estudo buscou o impacto de fatores de risco, como gênero, idade, tabagismo e DM, no padrão de distribuição anatômica das obstruções ateroscleróticas crônicas. A aterosclerose é a principal causa de DCVs e vem sendo estudada como uma doença inflamatória crônica, onde múltiplos fatores de risco interagem de maneira complexa com a parede arterial^28^ . A literatura sugere que mecanismos moleculares complexos estejam interagindo na formação e distribuição das diferentes lesões ateroscleróticas nos diversos territórios^18^ , o que mostra a importância de estudar a epidemiologia da DAP em cada região e em diferentes países.

## CONCLUSÃO

Concluindo, os fatores de risco para aterosclerose gênero, DM e tabagismo influenciam no padrão de distribuição anatômica da DAP em pacientes com isquemia crônica crítica de membros inferiores. O território femoropoplíteo foi o nível mais comum de oclusão proximal entre todos os grupos de pacientes, independentemente dos fatores de risco. Os doentes diabéticos apresentaram maior chance de que as oclusões pela DAP acometam apenas o território infragenicular. As mulheres tiveram maior chance de apresentar DAP com obstrução do território femoropoplíteo. Os pacientes tabagistas apresentam maior risco de comprometimento obstrutivo pela DAP no território aortoilíaco.
